# NKG2D ligands mediate immunosurveillance of senescent cells

**DOI:** 10.18632/aging.100897

**Published:** 2016-02-13

**Authors:** Adi Sagiv, Dominick G. A. Burton, Zhana Moshayev, Ezra Vadai, Felix Wensveen, Shifra Ben-Dor, Ofra Golani, Bojan Polic, Valery Krizhanovsky

**Affiliations:** ^1^ Department of Molecular Cell Biology, Weizmann Institute of Science, Rehovot, Israel; ^2^ School of Medicine, University of Rijeka, Croatia; ^3^ Bioinformatics and Biological Computing Unit, Weizmann Institute of Science, Rehovot, Israel; ^4^ Biological Services Department, Weizmann Institute of Science, Rehovot, Israel; ^5^ Present address: School of Life and Health Sciences, Aston University, Birmingham, UK

**Keywords:** senescence, DNA damage response, fibrosis, Natural Killer Cell, NKG2D, ERK

## Abstract

Cellular senescence is a stress response mechanism that limits tumorigenesis and tissue damage. Induction of cellular senescence commonly coincides with an immunogenic phenotype that promotes self-elimination by components of the immune system, thereby facilitating tumor suppression and limiting excess fibrosis during wound repair. The mechanisms by which senescent cells regulate their immune surveillance are not completely understood. Here we show that ligands of an activating Natural Killer (NK) cell receptor (NKG2D), MICA and ULBP2 are consistently up-regulated following induction of replicative senescence, oncogene-induced senescence and DNA damage - induced senescence. MICA and ULBP2 proteins are necessary for efficient NK-mediated cytotoxicity towards senescent fibroblasts. The mechanisms regulating the initial expression of NKG2D ligands in senescent cells are dependent on a DNA damage response, whilst continuous expression of these ligands is regulated by the ERK signaling pathway. In liver fibrosis, the accumulation of senescent activated stellate cells is increased in mice lacking NKG2D receptor leading to increased fibrosis. Overall, our results provide new insights into the mechanisms regulating the expression of immune ligands in senescent cells and reveal the importance of NKG2D receptor-ligand interaction in protecting against liver fibrosis.

## INTRODUCTION

Although once thought of as passive state of cell cycle arrest, senescent cells are now known to govern physiological processes such as tumor prevention and tissue repair [1-5]. The senescent state can be induced in response to various stressors leading to irreversible cell cycle arrest, and often the development of an altered secretome. Such stressors include telomere dysfunction, oncogene activation, direct DNA damage, elevated intracellular reactive oxygen species (ROS) and cell-cell fusion [1-3, 6]. Many triggers of the senescent program are associated with a persistent DNA damage response (DDR), consequently leading to activation of ataxia telangiectasia mutated (ATM) and ataxia telangiectasia and RAD3-related (ATR) [7-9]. ATM and ATR can inhibit cell cycle progression by promoting p53 accumulation, which subsequently regulates a number of target genes, including cyclin-dependent kinase inhibitor p21 (CDKN1A). Senescent cells also undergo significant changes in their chromatin organization leading to activation of pRb-p16 pathway that acts in parallel with the DDR-p53 pathway to induce and sustain the cell cycle arrest [10, 11]. Therefore, induction of senescence regulated by multiple signaling pathways performs essential physiological functions by limiting tumorigenesis and tissue damage.

Whereas the transient appearance of senescent cells plays important physiological roles, long-term persistence of senescent cells may promote pathological conditions via continuous secretion of pro-inflammatory factors [2, 4, 12, 13]. The secretion of chemoattractants and the up-regulation of adhesion molecules also forms part of an immunogenic phenotype often attributed to senescent cells [14, 15]. This phenotype promotes self-elimination of senescent cells by the immune system and can prevent the long-term negative effects stimulated by their presence [1, 14, 16]. The interaction of senescent cells with the immune system is necessary to fulfill the physiological functions of senescent cells and to prevent the long-term negative consequences of their presence.

One of the main physiological functions of senescent cells is tumor suppression [17, 18]. Immuno-surveillance of senescent cells facilitates this effect. Following tumor suppressor reactivation, tumor cells become senescent and are targeted by components of the innate immune system [19]. Following expression of mutant N-RAS, pre-malignant senescent hepatocytes undergo immune clearance mediated by an adaptive immune response [20]. Impaired immune surveillance of these hepatocytes promoted hepatocellular carcinomas in mice, demonstrating the importance of immunosurveillance of senescent cells as a tumor suppressor function.

In addition to its role in tumor suppression, immune clearance of senescent cells is necessary to limit short term tissue damage response [21, 22]. Induction of cell senescence in activated hepatic stellate cells limited liver fibrosis following injury. Subsequently, senescent cells are eliminated by Natural Killer (NK) cells to ensure proper reversion of fibrosis [21]. To eliminate senescent cells NK cells utilize granule exocytosis, but not death-receptor mediated pathway which is inhibited in senescent cells by DCR2 [23]. The granule exocytosis pathway is necessary for clearance of senescent cells in fibrotic livers and therefore for restraining fibrosis development. NK cell mediated immune surveillance is therefore necessary for the immunosurveillance of senescent cells during tumorigenesis, tumor therapy and tissue damage.

NK cells are a component of the innate immune system that rapidly responds to virally infected and tumor cells [24, 25]. One of the receptors responsible for the activation of the NK cells, NKG2D (NCBI symbol KLRK1) receptor, is implicated in interaction of NK cells with senescent cells during tumorigenesis and tumor therapy [26, 27]. NKG2D receptor recognizes ligands MICA/B and ULBP1-6 on the surface of stressed cells leading to direct cytotoxicity [24, 28, 29].

To date, our understanding concerning the interaction of senescent cells with NK cells comes primarily from a limited number of studies which focused on senescence induction in cancer cells rather than physiologically normal cells [26, 27]. Senescent cells derived from such normal cells accumulate at sites of tissue damage and during ageing. Therefore, we investigated the mechanisms regulating the interaction of senescent cells derived from normal cells with NK cells. We show that senescent cells induced by various stimuli present NKG2D ligands MICA and ULBP2 on their cell surface, enabling their recognition and elimination by NK cells. The expression of the ligands is regulated by a DNA damage response and ERK signaling. NKG2D receptor knockout in mice prevents efficient elimination of senescent cells during liver damage, leading to increased liver fibrosis. Overall, our study provides new insights into the mechanisms of immuno-surveillance of senescent cells and their regulatory pathways.

## RESULTS

### Expression of NKG2D ligands is elevated in senescent cells

**Figure 1:**
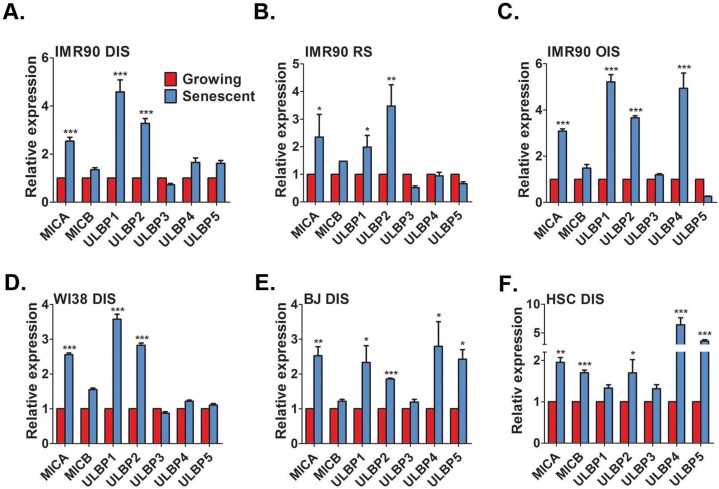
Senescent cells upregulate NKG2D ligands RT-PCR analysis demonstrates a consistent up-regulation of MICA, ULBP1, and ULBP2 in DNA damage-induced senescent (DIS) (**A**), replicative senescent (RS) (**B**), and H-RAS^v12^-mediated oncogene-induced senescent (OIS) (**C**) IMR-90 fibroblasts compared to growing (control) cells. Similarly, DIS WI38 (**D**) and BJ (**E**) fibroblasts and DIS hepatic stellate cells (HSCs) (**F**) also show an up-regulation of NKG2D ligands. The graphs represent the mean and the S.E.M of at least triplicate measurements from at least four independent experiments. Two-tailed t-test *P<0.05, **P<0.001, ***P<0.0001.

NK cell mediated immune surveillance of senescent cells limits tumorigenesis and tissue damage. To fulfill this function NK cells preferentially induce cell death in senescent cells via the granule exocytosis pathway [23]. We aimed to determine the mechanisms by which NK cells can specifically recognize senescent cells, leading to activation of the specific cytotoxicity towards senescent cells. NK cells express NKG2D receptors, which were previously implicated in interaction between NK cells and damaged cells, including cancer cells undergoing senescence in response to p53 reactivation or chemotherapy [19, 26, 27]. We therefore evaluated whether cells undergoing senescence in response to classical stimuli trans-criptionally upregulate common NKG2D ligands. In human cells these ligands are ULBP1-6, MICA and MICB. Normal human fibroblast IMR-90 cells were induced to senesce via direct DNA damage through treatment with etoposide (DIS), replicative senescence (RS) associated with telomere shortening by extended cell culture and oncogene induced senescence (OIS) via overexpression of H-RAS^v12^. Cell senescence was confirmed using several common biomarkers (Fig S1) and the expression level of the different NKG2D ligands were assessed. The NKG2D ligands MICA, ULBP1 and ULBP2 were consistently upregulated at least two-fold in all three types of senescent IMR-90 fibroblasts compared to growing control cells (p<0.05; Fig 1A,B,C for DIS, RS and OIS cells, respectively). Of note, in addition to MICA, ULBP1 and ULBP2, a five-fold increase in ULBP4 expression was observed in OIS cells but not DIS or RS cells in comparison to growing cells (p<0.0001; Fig 1C). Therefore, senescent IMR-90 cells upregulate mRNA levels of several ligands of NKG2D receptor.

To exclude the possibility that the expression of MICA, ULBP1 and ULBP2 was specific to IMR-90 fibroblasts, the expression of these ligands was determined in additional fibroblasts strains, namely WI38 and BJ, which were induced to senesce via DNA damage treatment. Similarly to IMR-90 cells, senescent WI38 and BJ fibroblasts significantly elevated the expression of MICA, ULBP1 and ULBP2 compared to growing cells (p<0.05; Fig 1D,E for WI38 and BJ, respectively). However, senescent BJ cells also upregulated the expression of ULBP4 and ULBP5 over two-fold compared to growing cells (p<0.05; Fig 1E). To evaluate the expression of the ligands in a physiologically relevant cell type, we assessed the expression of NKG2D ligands in activated hepatic stellate cells (HSCs). These cells become senescent during liver fibrosis and are essential in limiting the fibrotic response [21]. In concordance with the previous results, a significant two-fold elevation in MICA and ULBP2 was observed compared to growing activated HSCs (p<0.05; Fig 1F). However, senescent cells derived from activated HSCs did not upregulate ULBP1 (Fig. 1F). In addition, senescent activated HSCs elevated MICB, ULBP4 and ULBP5 compared to growing cells (p<0.0001 each; Fig 1F). Overall, these findings demonstrate that senescent cells upregulate the expression of NKG2D ligands. Importantly, this upregulation occurs regardless of the senescence-inducing stimuli, and the exact expression profile of these NKG2D ligands can differ between cell strains and cell types.

### MICA and ULBP2 proteins are up-regulated and localized to the cell surface of senescent cells

**Figure 2:**
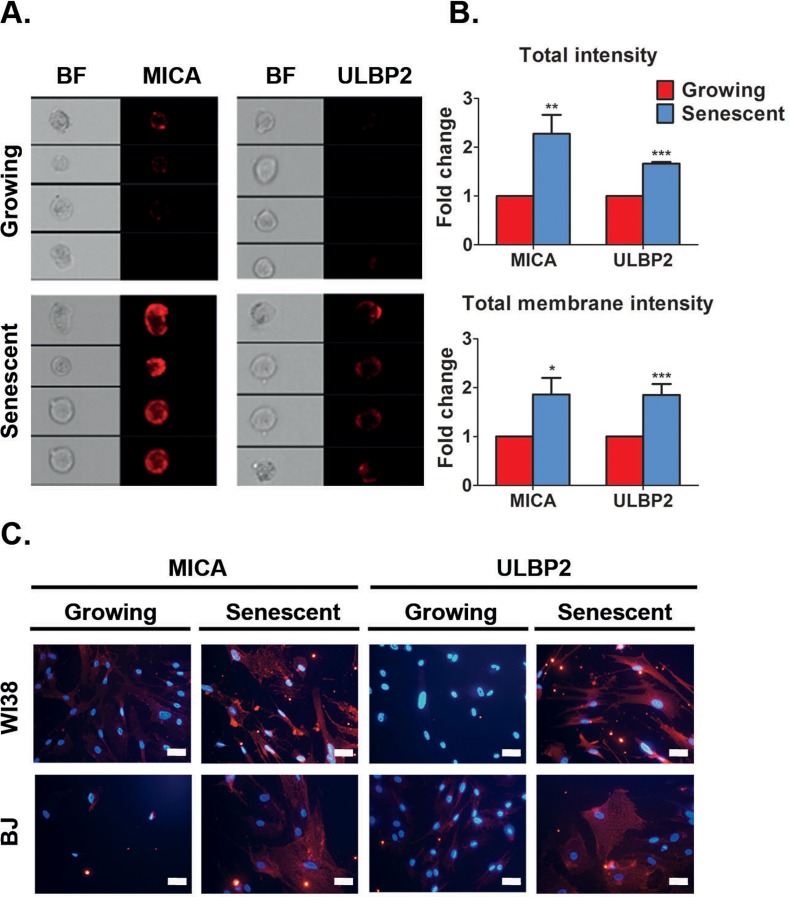
Senescent cells upregulate MICA and ULBP2 on the cell surface (**A**) ImageStream analysis demonstrates higher expression levels of MICA and ULBP2 on the cell surface membrane of DIS IMR-90 fibroblasts (Senescent) compared to control (Growing) cells. (**B**) Quantification of total intensity and total membrane intensity indicate higher levels of MICA and ULBP2 in DIS IMR-90 cells compared to growing (control) cells. (**C**) Representative immunofluorescence staining of MICA and ULBP2 performed on non-permeabilized cells demonstrates higher expression levels of these proteins on the cell surface membrane in DIS WI38 and BJ cells compared to growing (control) cells. Scale bar: 50μm. MICA and ULBP2 are shown in red. DAPI is shown in blue. Data presented as mean with S.E.M of three independent experiments. Two-tailed t-test *P<0.05, **P<0.001, ***P<0.0001.

Localization of ligands to the plasma membrane allows interaction of target cells with immune cells. Therefore, we wanted to determine whether NKG2D ligands are localized to the cell surface membrane of senescent cells utilizing DIS cells as a proof of principle model. To this end, we focused on MICA and ULBP2 localization, since they are consistently upregulated at the mRNA level in all senescent cells we evaluated, particularly in HSCs, a physiologically relevant cell type (Fig 1). We used the ImageStreamX system, that combines imaging and flow cytometry, to monitor the intensity and localization of the MICA and ULBP2 proteins (Fig 2A).

Total and membrane bound intensity of MICA or ULBP2 proteins in DIS IMR-90 cells was subsequently measured. This analysis revealed that total intensity of MICA and ULBP2 was elevated two-fold (p<0.001 for MICA and p<0.0001 for ULBP2; Fig 2B), and the intensity of the protein on the cell membrane was also elevated two-fold in senescent cells compared to growing cells (p<0.05 for MICA and p<0.0001 for ULBP2; Fig 2B). Of note, performing standard flow cytometry analysis on DIS IMR-90 cells also confirmed MICA and ULBP2 protein upregulation on the cell surface membrane (Fig S2A). Increased protein abundance of MICA and ULBP2 was also observed in DIS WI38, BJ and IMR90 cells compared to growing cells as determined by fluorescent intensity via immunocytochemistry (Fig 2C and Fig S2B). These findings demonstrate that the transcriptional upregulation of MICA and ULBP2 is subsequently correlated with an elevation in protein abundance, localized at the cell membrane where they can be potentially identified by NK cells.

### MICA and ULBP2 facilitate NK cell mediated cytotoxicity towards senescent cells

Following the presentation of NKG2D ligands on the cell surface of stressed cells, NK cells expressing NKG2D receptors can recognize and interact with these cells to promote specific cytotoxicity [24, 30]. We have previously showed that NK cells exhibit specific cytotoxicity towards senescent cells *in vitro* [21, 23].

**Figure 3:**
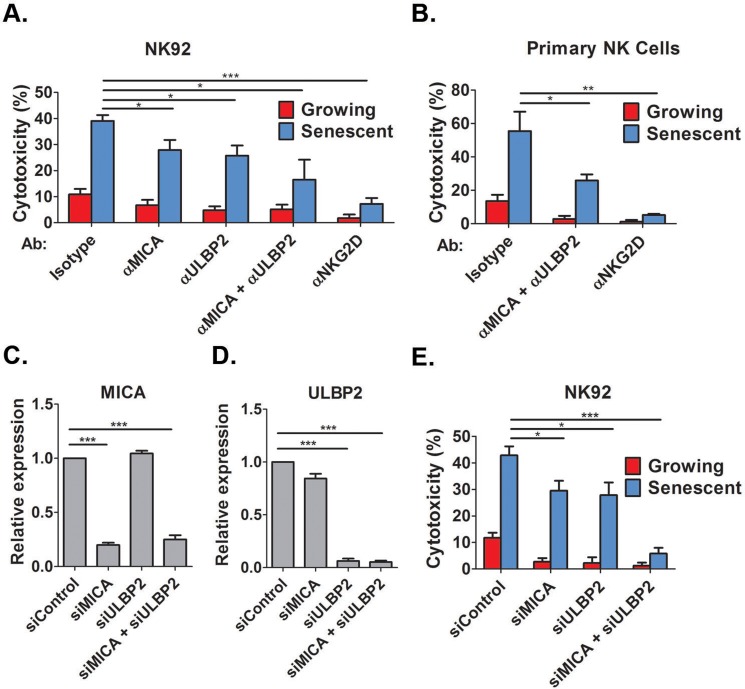
NKG2D receptor-ligand interaction mediates the recognition of senescent cells by NK cells Growing and senescent (DIS) IMR-90 fibroblasts were co-incubated with either NK92 (**A**) or primary NK cells (**B**) in the presence of different blocking antibodies and the percent of cytotoxicity towards senescent cells was assessed after 2 hours. (**C**,**D**) MICA and ULBP2 were knocked down using specific siRNA and knockdown efficiency was evaluated by quantitative RT-PCR. (**E**) The degree of cytotoxicity of NK92 cells towards senescent cells was assessed following the knockdown of MICA and ULBP2 in senescent or growing (control) cells. Data presented as mean with S.E.M of three independent experiments. *P<0.05, **P<0.001, ***P<0.0001.

Of note, our cytotoxicity methodology quantifies the remaining viable cells at the end of the co-incubation period using a viability assay. Traditional NK-mediated cytotoxicity assays that rely on the loading of the target cells with ^51^Cr cannot be applied when using senescence cells since efficient loading requires a threshold level of cell proliferation [31] which cannot be achieved in senescent cells. NKG2D ligands are present on the membrane of senescent cells (Fig 2), and we now aimed to determine whether these ligands are required for NK cell mediated cytotoxicity towards senescent fibroblasts. We treated DIS senescent IMR-90 cells with blocking antibodies against MICA and ULBP2 and incubated these cells with either the NK92 NK cell line (Fig 3A) or primary human NK cells (Fig 3B) and assessed the degree of cytotoxicity. Blocking antibodies against either MICA or ULBP2 alone reduced NK92 mediated cytotoxicity towards senescent cells by 25% (p<0.05; Fig 3A), whereas combined inhibition of MICA and ULBP2 reduced cytotoxicity by NK92 and primary NK cells to less than a half comparing to isotype control antibody (p<0.05; Fig 3A,B). To evaluate the contribution of the NKG2D receptor itself for the recognition of senescent cells, we blocked the NKG2D receptors on NK cells using blocking antibodies prior to co-culture with senescent cells. Blocking of the receptor significantly reduced the cytotoxicity towards senescent cells by both NK92 and primary NK cells (80%, p<0.001 for NK92 and 90%, p<0.0001 for primary NK ; Fig 3A,B). Therefore, blocking the interaction between MICA, ULBP2 and their receptor NKG2D significantly reduces the NK cell mediated cytotoxicity towards senescent cells.

To evaluate the effect of the ligands on the recognition of senescent cells by NK cells using an independent approach, the expression of MICA and ULBP2 was down-regulated using specific siRNA mixes. The siRNAs induced at least 75% knockdown of MICA and ULBP2 as was confirmed by quantitative RT-PCR (p<0.0001, Fig 3C and D for MICA and ULBP2, respectively). Knockdown of either MICA or ULBP2 alone reduced NK92 mediated cytotoxicity by one third (p<0.05; Fig 3E), whereas combined knockdown of MICA and ULBP2 completely blocked the cytotoxicity of NK cells towards DIS cells (p<0.0001; Fig 3E). Therefore, expression of MICA and ULBP2 in senescent cells is necessary for the NK mediated cytotoxicity towards these cells. Overall, these findings demonstrate that NKG2D receptor-ligand interaction is essential for NK mediated killing of senescent cells.

### DNA damage response upregulates expression of ULBP2, but not MICA

**Figure 4:**
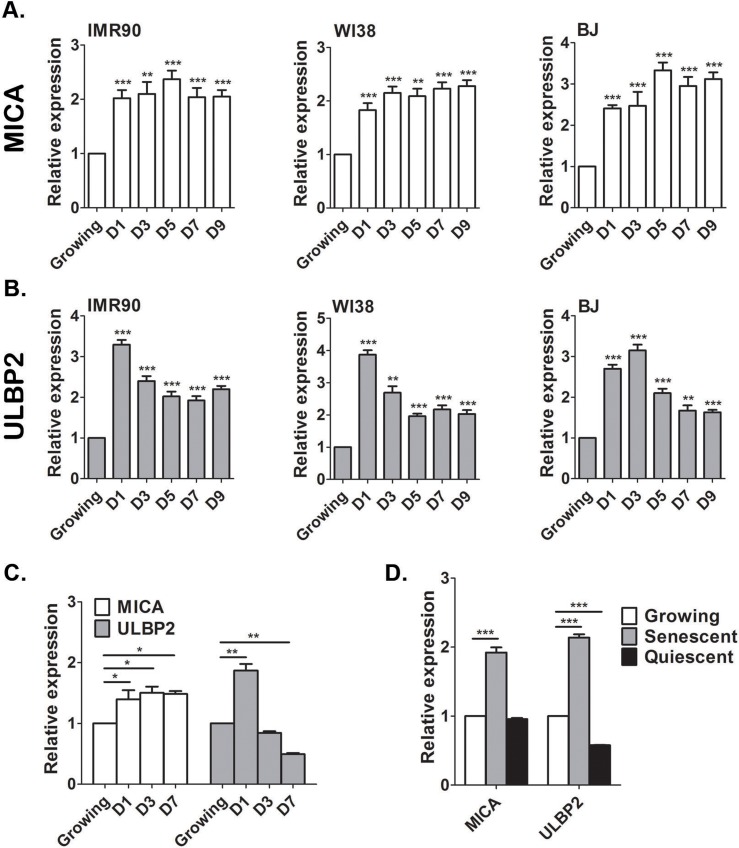
MICA and ULBP2 expression is stably upregulated in senescent, but not quiescent cells Growing IMR-90, WI38 and BJ cells were treated with Etoposide (100μM) and the level of MICA and ULBP2 expression were assessed by RT-PCR. The expression of MICA (**A**) and ULBP2 (**B**) was elevated 24 hours following treatment and was maintained at higher levels throughout the indicated time points. Growing cells at day 0 served as control. (**C**) Growing cells were treated with a low concentration of Etoposide (10μM) to induce transient growth arrest and the level of MICA and ULBP2 expression were assessed by RT-PCR. (**D**) IMR-90 cells were grown to confluence and maintained for a further 7 days until cells became quiescent. Cell-cycle arrest induced by quiescence was not sufficient to elevate MICA and ULBP2 expression as assessed by RT-PCR and compared to growing and senescent cells. Data presented as mean with S.E.M of three independent experiments. Two-tailed t-test *P<0.05, **P<0.001, ***P<0.0001.

To understand the regulation of the interaction between senescent cells and NK cells, we aimed to underpin the mechanisms that promote transcriptional upregulation of NKG2D ligands during induction of senescence. Importantly, we observed a correlation between the levels of MICA and ULBP2 mRNA transcripts and the levels of protein expression on the cell surface membrane in senescent cells (Fig. 1A and Fig. 3). Therefore, we decided to focus our studies on the molecular mechanisms regulating mRNA expression of NKG2D ligands in senescent cells. NKG2D ligands can be upregulated in response to different forms of cellular stress, including DNA damage [27, 32]. DDR is activated in senescent cells and therefore we wanted to understand the contribution of this pathway to the regulation of MICA and ULBP2 expression. The upregulation of the ligands following genomic stress could either be an early response that is subsequently maintained when cells enter senescence or be a senescence specific response that occurs at later time points. To distinguish between the two possibilities we determined the expression dynamics of MICA and ULBP2 over a nine-day period leading to a full senescent phenotype in IMR-90, WI38 and BJ cells. The expression of MICA (Fig 4A) and ULBP2 (Fig 4B) was determined at days 0, 1, 3, 5, 7 and 9 following treatment of proliferating cells with 100μM etoposide. The expression of MICA is elevated at least by two-folds within 24hrs of etoposide treatment and is maintained consistently high throughout the nine days in all cell strains (p<0.001; Fig 4A). ULBP2 expression has a slightly different expression pattern associated with an initial elevation in expression by about three-fold within the first 24hrs, which subsequently decreased but was maintained consistently upregulated about two-fold (p<0.001) throughout the nine days compared to non-senescent control cells (Fig 4B). In contrast, cells treated with 10μM etoposide to induce transient DNA damage and cell cycle arrest rather than cell senescence demonstrated an initial one and a half-fold upregulation of MICA (p<0.05, Fig 4C) and two-fold upregulation of ULBP2 (p<0.001). By day 3, ULBP2 levels decrease by 50% compared to growing cells following treatment with 10μM etoposide for 24hrs (p<0.001, Fig 4C), while MICA levels remain stable. Therefore, short-term DNA damage can activate transient upregulation of NKG2D ligands.

To determine whether the upregulation of MICA and ULBP2 is a response to cell cycle arrest or rather a response to DNA damage, IMR-90 cells were grown to confluence and cells were maintained for a further 7 days to induce quiescence via contact inhibition, a growth arrested state absent of DNA damage. Quiescence was validated by the absence of the proliferation marker Ki67 (Fig S3) and the ability to re-enter the cell cycle when subsequently sub-cultured. Strikingly, quiescent IMR-90 cells do not upregulate the expression of MICA or ULBP2, but rather downregulate ULBP2 expression (50%, p<0.0001) compared to proliferating controls (Fig 4D). Therefore, cell cycle arrest by itself does not contribute to the upregulation of the expression of the NKG2D ligands.

To assess whether the upregulation of MICA and ULBP2 expression is regulated by DDR, proliferating IMR-90 cells were pretreated with the ATM inhibitor (KU60019) for one hour prior to the addition of 100μM etoposide. Expression level of MICA and ULBP2 was evaluated twenty four hours following the treatment.

**Figure 5:**
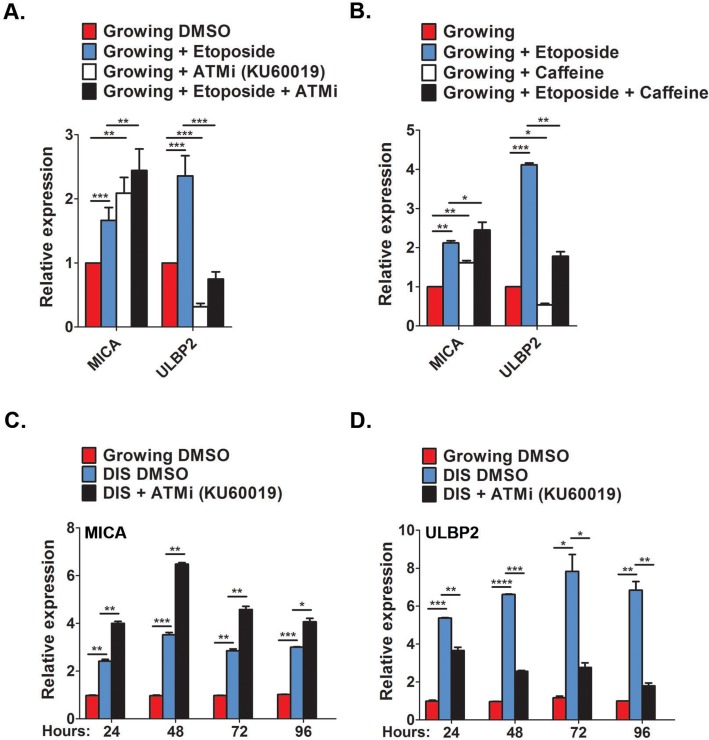
Contribution of DDR for MICA and ULBP2 expression (**A**,**B**) Growing cells were treated with either the ATM inhibitor KU60019 (10μM, A) or the ATM/ATR inhibitor Caffeine (5mM, B) for 1 hour prior to Etoposide treatment (100μM). Twenty four hours later the level of MICA and ULBP2 expression were assessed by RT-PCR. (**C**,**D**) DIS IMR-90 cells were treated with KU60019 (10μM) for 24, 48, 72 and 96 hours and the expression of MICA (**C**) and ULBP2 (**D**) was assessed at each time point via RT-PCR. Data presented as mean with S.E.M of three independent experiments. Two-tailed t-test *P<0.05, **P<0.001, ***P<0.0001.

Cells pretreated with the ATM inhibitor did not upregulate expression of ULBP2 whilst MICA is still upregulated compared to etoposide only treated cells (p<0.0001; Fig 5A). To evaluate the possibility that MICA may instead be regulated via ATR, or by both arms of the DDR including ATM and ATR, we performed the experiments using caffeine, an inhibitor of both ATM and ATR. Pre-treatment with caffeine prevented upregulation of ULBP2, whereas a further upregulation was observed with MICA expression (p<0.05; Fig 5B). To determine the regulation of MICA and ULBP2 expression in cells that have already been established as senescent, DIS IMR-90 fibroblasts were treated with the ATM inhibitor for 24, 48, 72 and 96 hours with daily replenishments. ATM inhibition caused a 25% reduction in ULBP2 expression within twenty four hours (p<0.001), with further reductions at later time points (>50%, p<0.0001, p<0.05 and p<0.001 for 48, 72 and 96 hours, respectively; Fig 5C,D).

However, ATM inhibition also further increased MICA expression throughout all time points observed (p< 0.001 for 48 and 72 hours and p<0.05 for 96 hours; Fig 5C,D). Therefore, upregulation of ULBP2 during cell senescence appears to be dependent upon a DNA damage response, whereas the upregulation of MICA is controlled by mechanisms independent of DNA damage.

### ERK activity regulates the expression of MICA and ULBP2

**Figure 6:**
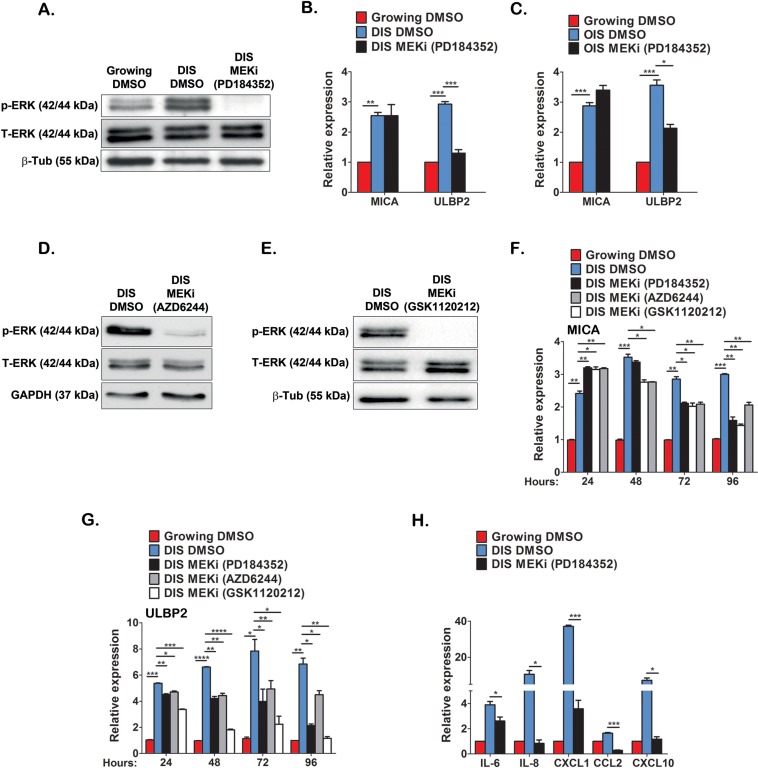
ERK activity regulates MICA and ULBP2 expression in DIS cells Western blot analysis was performed on lysates from DIS IMR-90 cells and growing cells to evaluate ERK1/2 phosphorylation in senescent cells and to assess the effect of PD184352 (10μM, 48 hours) on ERK1/2 phosphorylation (**A**). Total ERK and tubulin expression were evaluated as controls. (**B**,**C**) The expression level of MICA and ULBP2 was determined following inhibition of ERK activity for 48 hours on DIS (**B**) and OIS (**C**) cells. Two additional inhibitors of MEK (AZD6244 and GSK1120212) also reduced ERK1/2 phosphorylation (**D** and **E**) and significantly reduced the expression of MICA and ULBP2 over indicated incubation periods as assessed by RT-PCR (**F** and **G**). PD184352 also significantly reduced the expression of indicated cytokines as assessed by RT-PCR (**H**). Data presented as mean with S.E.M of three independent experiments. Two-tailed t-test *P<0.05, **P<0.001, ***P<0.0001.

One of the signaling pathways constitutively activated during cell senescence is ERK (Fig 6A and [33]). In order to determine additional molecular regulators governing the expression of immune ligands in senescent cells, we analyzed the role of ERK signaling in regulating expression of NKG2D ligands. Phosphorylation of ERK increases its activity and MEK is the kinase that phosphorylates and activates ERK. In order to evaluate the impact of ERK signaling on expression of NKG2D ligands in senescent cells, DIS and OIS IMR-90 cells were maintained in the presence of a MEK inhibitor (PD184352) for 48 hours. Western blot analysis confirmed inhibition of ERK phosphorylation in this setting (Fig 6A). We then assessed the expression of MICA and ULBP2 in senescent cells treated with PD184352 or a vehicle control (DMSO) and in growing cells. In the presence of PD184352, no change in the expression of MICA was observed, whereas a substantial decrease (>50%) in ULBP2 expression was observed within DIS and OIS cells (p<0.0001 and p<0.05 for DIS and OIS, respectively; Fig 6B,C). Similarly, ERK inhibition in DIS WI38 cells caused ULBP2 downregulation with no impact on MICA expression (Fig S4). In addition to PD184352, two additional MEK inhibitors (AZD6244 and GSK1120212) were evaluated for their effect on MICA and ULBP2 expression in senescent cells over 24, 48, 72 and 96 hours (Fig 6D,E). Whilst these MEK inhibitors caused a mild elevation in MICA expression within the first 24hrs, at later time points they lead to a 25% to 50% reduction in MICA expression, particularly by 72 and 96hrs following treatment (p<0.05; Fig 6F). These inhibitors caused a 20% reduction in ULBP2 within 24 hours and further reductions between 25% and 60% at later time points (p<0.05; Fig 6G). To rule out the possibility that decreases in ligand expression at later time points was due to cells dying rather than ERK inhibition *per se*, cell viability was determined at each time point. No significant difference in cell viability was observed in cells treated with MEK inhibitors compared to DMSO treated cells (Fig S5). Therefore, ERK signaling is essential for the continuous expression of MICA and ULBP2 in senescent cells.

In addition to NKG2D ligands, the immune surveillance of senescent cells by NK cells is also regulated by other immune ligands as well as the senescent secretome, functioning to attract immune cells to the sites of senescent cells [14, 25]. Interestingly, ERK inhibition in DIS IMR-90 cells downregulated PVR but not ICAM-1 (Fig S6), two immune modulators that are implicated in NK-mediated cytotoxicity [34, 35]. Furthermore, ERK inhibition promotes downregulation in the expression of components of the senescent secretome (IL-6, IL-8, CXCL1, CXCL10 and CCL2) that can act as chemoattractants for immune cells (Fig 6H). These findings suggest that ERK activity in senescent cells is important for their interaction with NK cells by regulating the expression of NKG2D ligands, specifically MICA and ULBP2, and the expression of chemoattractants.

### NKG2D is required for NK cell mediated immune surveillance of senescent cell *in vivo*

**Figure 7:**
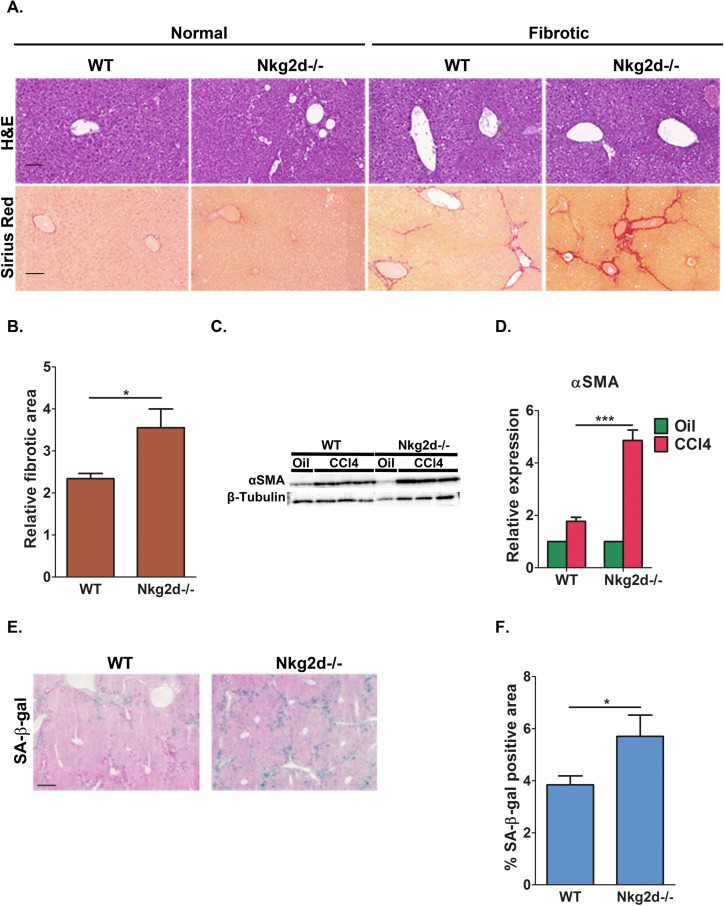
NKG2D receptor promotes senescent cell clearance and limits liver fibrosis *in vivo* *Nkg2d* knockout (Nkg2d*^−/−^*) and WT mice were treated with CCl_4_ to induce fibrosis. (**A**) H&E and Sirius red staining was undertaken on liver sections. Scale bar: 100μm. (**B**) Quantification of the fibrotic area demonstrate increased fibrosis in *Nkg2d^−/−^* livers compared to WT following CCl_4_ treatment. (**C**) Expression of αSMA, a marker of activated HSCs, was determined by Western blot analysis of whole-liver extracts and (**D**) by quantitative RT-PCR. Four mice of each genotype are shown for (**C**). (**E**) SA-β-gal staining identified accumulation of senescent cells along the fibrotic scar areas in the livers of WT and *Nkg2d^−/−^* mice. Scale bar: 100μm. (**F**) Quantification of the SA-β-gal staining of the livers of WT and *Nkg2d^−/−^* mice. At least six mice of each genotype were used for the quantitative analysis in **B** and **F**; Two-tailed t-test *P<0.05, ***^P^<0.0001.

In response to tissue damage within the liver, hepatic stellate cells (HSCs) become activated and undergo proliferative expansion that is associated with deposition of extracellular matrix (ECM) components [36]. To prevent excess fibrosis, activated HSCs undergo senescence and are subsequently eliminated by NK cells [21]. We wanted to determine whether NKG2D receptor-ligand interactions are required for NK-mediated immune clearance of senescent cells *in vivo* in liver fibrosis. Importantly, murine Nkg2d-ligands were shown to be upregulated in fibrotic livers of WT mice and to mediate NK killing of activated HSCs in an Nkg2d-dependent manner thereby ameliorating liver fibrosis in these mice [37]. To evaluate this we induced fibrosis in wild-type (WT) and *Nkg2d* knockout (*Nkg2d^−/−^*) C57BL/6 mice by 12 consecutive bi-weekly injections of CCl4. To assess fibrosis progression, liver sections from CCl4 and vehicle treated mice were stained with Hematoxylin and Eosin (H&E) (Fig 7A). Fibrosis was not detected in vehicle treated mice, nor were there any detectable differences in liver architecture between WT and *Nkg2d^−/−^* mice. As expected, livers from CCl4-treated mice of both genotypes displayed a characteristic fibrotic histology (Fig 7A). The fibrosis is characterized by activation of HSCs and deposition of extracellular matrix (ECM) by these cells. The amount of activated HSCs and the amount of ECM deposited in the fibrotic scar are criteria for evaluation of the progression of fibrosis [36]. We therefore evaluated the ECM deposition and the amount of HSCs in fibrotic livers of WT and *Nkg2d^−/−^* mice. Fibrotic scars were identified by Sirius Red staining and quantified using morphometric analysis of whole-liver sections. The fibrotic scar area is increased by 50% in *Nkg2d^−/−^* mice comparing with WT (p<0.05; Fig 7A,B). The relative abundance of activated HSCs was assessed by expression of the HSC marker αSMA (alpha smooth muscle actin) in whole-liver lysates. Immunoblotting and RT-PCR analysis revealed more than two-fold increase in mRNA levels of αSMA and a corresponding increase in its protein level in CCl4-treated *Nkg2d^−/−^* livers compared with WT controls (p<0.0001; Fig 7C,D). These results indicate an increase in fibrosis and impaired elimination of HSCs in *Nkg2d^−/−^* mice.

To determine whether the NKG2D-ligand interactions influences the abundance of senescent cells, we examined *Nkg2d^−/− and WT^* fibrotic livers for the senescent marker SA-β-gal following CCl_4_ treatment. Consistent with our previous reports [21, 23], SA-β-gal positive cells were found predominantly in the livers of CCl_4_-treated mice and in areas adjacent or within the fibrotic scars (Fig 7E). Quantitative imaging of the tissue area occupied by SA-β-gal-positive cells showed a 45% increase in senescent cells retained in *^Nkg2d−/−^* livers compared to control mice (p<0.05; Fig 7E,F). In summary, these findings demonstrate that NKG2D receptor-ligand interaction is important for the elimination of senescent cells *in vivo* and that lack of NKG2D impairs immune clearance of senescent cells, thereby contributing to excess tissue fibrosis following liver damage.

## DISCUSSION

Senescent cells are specifically recognized and eliminated by NK cells [19, 21, 23, 26, 27]. In this study we investigated the mechanisms which control the recognition of senescent cells by NK cells. We found that senescent cells up-regulate the expression of NKG2D ligands MICA and ULBP2 regardless of the senescence-inducing stimuli. The mechanisms regulating the expression of NKG2D ligands in senescent cells are partly attributed to a DNA damage response and activation of ERK activity. MICA and ULBP2 were found to be localized at the cell membrane where they can interact with NK cells to mediate efficient killing of senescent cells. Interaction of the ligands with the NKG2D receptor on the NK cells is necessary for the recognition of senescent cells by the NK cells *in vitro*. Importantly, NKG2D receptor-ligand interaction is essential for efficient elimination of senescent cells *in vivo* and thus for restraining fibrosis development. Overall, our findings demonstrate that NKG2D ligands on senescent cells are necessary for efficient recognition and elimination of senescent cells *in vitro* and during tissue damage *in vivo*.

The increase in expression of NKG2D ligands, particularly MICA and ULBP2, is likely a general feature of human senescent cells. A number of other studies have demonstrated the expression of MICA and/or ULBP2 in senescent cells derived from different cell types, namely human activated stellate cells [21], replicative senescent human umbilical vein endothelial cells [38] and chemotherapy-induced senescent multiple myeloma cells [27]. Furthermore, senescent cells also acquire unique NKG2D ligand expression profiles consisting of several additional NKG2D ligands that result from differences between cell types (or cell-strains) and the mechanism by which senescence was induced. The repertoire of NKG2D ligands in mice is vast and similar to human cells, however based on sequence comparisons, mouse ligands are not homologous to the human ligands [28, 39]. Of note, NKG2D ligands are present on mouse cells that become senescent following p53 reactivation, and participate in the interaction of these cells with NK cells [19, 26]. In addition to their expression in senescent cells, NKG2D ligands are upregulated in other cell contexts related to cellular stress, including cancer, virally infected cells or following DNA damage [24, 25, 28, 39]. Therefore, the expression of these ligands might be part of a general stress response of cells that is utilized by senescent cells.

The expression of NKG2D ligands in senescent cells is independent of cell cycle arrest and regulated by several pathways activated in senescent cells in parallel, rather than an individual pathway. This is supported by findings in mice demonstrating that quiescent hepatic stellate cells do not express the NKG2D ligand, RAE-1, whereas high levels are observed in activated stellate cells, a cell response associated with induction of fibrosis and senescence in this cell type [21, 37]. Similarly, the senescent secretome is independent of cell cycle arrest and regulated by interconnected pathways involving DDR, p38, NF-κB, IL-1β and mTOR [1, 40-46]. Various triggers of cellular senescence lead to activation of persistent DDR which could lead to the upregulation of NKG2D ligands we observed. Indeed, DNA damage was previously shown to induce expression of NKG2D ligands in normal mouse cells and human cancer cells [27, 32]. However, additional mechanisms in parallel with DDR likely also regulate the expression of ligands. For example, Ras can induce expression of Nkg2d ligands in mouse cells independent of DDR [47]. In addition, type I interferon's contribute to the expression of both MICA and ULBP2 once cells have become senescent [48]. Interestingly, IFN expression has also been shown to be induced by DNA damaging agents, consequently contributing to senescence induction by further amplifying the DNA damage responses [49]. Unexpected was the observation that inhibition of ATM elevated MICA expression but reduced ULBP2 expression, suggesting that MICA is suppressed rather than activated by DDR during senescence.

Another pathway that is active in senescent cells is extracellular signal-regulated kinase 1/2 (ERK1/2) cascade. ERK signaling is a central signaling pathway that regulates proliferation, survival, and also stress response [50]. However, several studies suggest that phosphorylated ERK localization maintains cytoplasmic, but not nuclear, within senescent cells [51-53]. Therefore, ERK could regulate NKG2D ligand expression in senescent cells by mechanisms independent of transcriptional regulation. Our findings suggest that the mRNAs coding for NKG2D ligands might be stable in senescent cells and therefore it is conceivable that ERK promotes mRNA stability via downregulation of miRNA activity. In fact, MICA and MICB have been reported to be regulated by endogenous miRNAs in tumors and following cytomegalovirus infection [39]. The longer time frame required to observe the same decline in MICA expression following ERK inhibition compared to ULBP2 expression may reflect differences in the regulation of mRNA stability between the two ligands. In addition to regulation of NKG2D ligands, ERK signaling in senescent cells regulates expression of chemokines that can serve as chemoattractants for NK cells and other immune cells. The expression of these cytokines is also regulated by the DDR [44]. This implies that multiple components of the senescent phenotype are responsible for their interaction with the immune system, regulated via the DDR and ERK signaling. It would be important to determine if other aspects of non-cell-autonomous interactions of stressed cells, like intercellular protein transfer [54], are also regulated by these pathways.

The accumulation of senescent cells in livers of *Nkg2d* knockout mice following tissue damage demonstrates the importance of the Nkg2d mediated recognition for the removal of senescent cells and prevention of excess fibrosis. These effects are similar to the ones observed in *Prf1* knockout mice, where cytotoxic ability of NK cells is impaired [23]. In addition to NK cells, T-cell subsets also display NKG2D receptors [28, 39], suggesting that immunosurveillance of senescent cells could be a combined function of both NK cells and T-cells. T-cells play an essential role in immuno-surveillance of hepatocytes induced to senescence by expression of mutant *N-RAS* [20]. However, this pathway of immunosurveillance is dependent on antigen presentation and therefore might be independent of NKG2D receptors. Another NKG2D independent immune system component involved in immuno-surveillance of senescent cells are macrophages. Therefore, immunosurveillance of senescent cells is a complex program and a combination of innate and adaptive immune system is responsible for the proper clearance of senescent cells from the organism. The efficient elimination of senescent cells might not only be necessary for restraining fibrosis and enhancing tumor suppression. Whilst it is not known why senescent cells accumulate in later life, it has been suggested that ageing of the immune system may lead to impaired elimination of senescent cells, thereby promoting their accumulation within tissues [15, 55]. The expression of NKG2D receptors on NK cells does not appear to alter with age, but decreases in NK activity have been reported [55]. Therefore, impaired elimination of senescent cells could lead to their accumulation in tissues in pathological conditions and ageing.

Our findings add to the emerging conceptual idea that the senescent program might represent a change in cell state that is associated with conversion to an immunogenic phenotype, functioning to remove damaged cells by immune clearance rather than through apoptosis. In addition to the upregulation of NKG2D ligands, the secretion of chemoattractants or the expression of adhesion molecules are further examples by which senescent cells become immunogenic. Immune clearance of senescent cells is likely beneficial in complex organisms where the regenerative capacity is dependent on non-resident stem cell populations and therefore temporal preservation of tissue architecture is necessary. Elimination of senescent cells following short-term insults, mediated by immune clearance, has physiological functions in tumor suppression and wound healing. Moreover, inefficient clearance might lead to the long-term persistence of senescent cells in tissues that has been associated with promotion of cancer development, ageing and age-related disease [2, 4, 12]. Therefore, understanding the normal processes and mechanisms by which senescent cells are eliminated by the immune system will enable the formulation of conjectures concerning the mechanism responsible for impaired senescent cells elimination in later life. Such an understanding could lead to novel therapeutic strategies that enhance elimination of senescent cells by the immune system to improve tissue repair, cancer therapy and prevent deleterious effects of accumulation of senescent cells.

## MATERIALS AND METHODS

### Tissue culture and cytotoxicity assays

Human diploid fibroblasts, IMR-90 and WI38 (ATCC, Manassas, VA, USA), human foreskin fibroblasts BJ (ATCC, Manassas, VA, USA) and primary human hepatic myofibroblasts (activated HSCs) (Dominion Pharmakine, Derio – Bizkaia, Spain) were grown in standard conditions (DMEM supplemented with 10% FCS, 1% L-Glutamine and 1% Penicillin-Streptomycin and kept at 37°C with 7.5% CO_2_). DNA damage-induced senescent cells were generated by treating growing cells with Etoposide (100 μM, Sigma) for 48 hours. Cells were considered senescent 7 days after Etoposide removal. Oncogene induced senescence was achieved by retroviral infection of IMR-90 cells with mCherry-H-Ras^v12^ or mCherry as control as previously described [54], and cells were considered senescent 9 days after the end of infection. NK-92 NK cell lines (ATCC, Manassas, VA, USA) were grown according to the ATCC instructions.

*In vitro* cytotoxicity assays using the NK cell line, NK-92, were performed as described previously [21]. Briefly, growing or DIS IMR-90 cells were plated in a 12-well plate at 5×10^5^ per well; 10×10^5^ NK-92 cells were subsequently added to each well. Following 2 hours of co-incubation, NK-92 cells were washed gently and the cytotoxicity was determined based on quantification of remaining adherent cells using Presto Blue (Life Technologies, CA, USA) according to the manufacturer's instructions.

For *in vitro* cytotoxicity assays performed with primary human NK cells (gift from O. Mandelboim, The Hebrew University Hadassah Medical School, Jerusalem, Israel), target cells were plated in 12-well plates at 4×10^4^ cells per well; 1×10^5^ NK cells (more than 99% of CD56^+^;CD3^−^ [56] were subsequently added to each well. Following 2 hours of co-incubation, primary NK cells were washed gently and the cytotoxicity was determined based on quantification of remaining adherent cells using Presto Blue (Life Technologies, Carlsbad, California) according to the manufacturer's instructions.

For *in vitro* cytotoxicity assays using blocking antibodies, growing or senescent cells were plated in 24 well plates at 2×10^5^ cells per well. Growing and senescent cells were treated with blocking antibodies against MICA, ULBP2, and a mix of the isotype controls IgG2A and IgG2B according to the manufacturer's instructions (R&D Systems, MN, USA). In brief, the blocking antibodies at the indicated concentrations were added to each well in 200μl of 10% complete RPMI (1% Glutamine, 1% Non-essential Amino Acids, 1% Pyruvate, 1% pen-strep and 10% FCS) and incubated for 40 minutes at 37°C. Subsequently, 5×10^5^ of either NK92 cells or primary NK cells were co-cultured with the treated target cells. Following 2 hours of co-incubation, NK92 or primary NK cells were washed gently and the cytotoxicity was determined based on quantification of remaining adherent cells using Presto Blue (Life Technologies, Carlsbad, California, USA) according to the manufacturer's instructions. When using the blocking antibody against NKG2D, NK92 or primary NK cells were pre-incubated in the presence of αNKG2D for 40 minutes, washed, and then added to either growing and senescent cells. The cytotoxicity assay was done as described above.

For *in vitro* cytotoxicity assays using siRNA against MICA and ULBP2. ON-TARGETplus SMARTpool small-interfering RNA targeting MICA, ULBP2 and the nontargeting (control) pool were transfected into growing IMR-90 cells with Dharmafect 1 reagent (all from Dharmacon, Lafayette, CO, USA) according to the manufacturer's instructions. 48 hr after transfections cells were treated with 100μM Etoposide (Sigma) for 48 hours. Subsequently, a second transfection was performed using siRNA against MICA, ULBP2 and the nontargeting (control) pool to ensure gene knockdown for 9 days until cells become fully senescent. Then, a cytotoxicity assay using NK-92 cell was performed as described above.

### Mice

The Nkg2d^−/−^ mice development and characterization was described previously [57]. C57Bl6 mice served as controls. For fibrosis induction, the mice were treated twice a week, with i.p. injection of 1 ml/kg CCl4, for 6 weeks as described [21]. Formalin-fixed paraffin-embedded tissues were sectioned and stained either with hematoxylin–eosin for routine examination, or with Sirius red for visualization of fibrotic deposition. For analysis of relative fibrotic area and SA-β-gal activity, Images were taken from multiple slides using 3D Histech Panoramic MIDI Digital slide scanner (3DHISTECH Kft., Budapest, Hungary). The relative fibrotic area was calculated using the Fiji software [58]. The Relative fibrotic area was calculated by dividing the total Sirius red area by the total analyzed area. At least 7 mice were analyzed from each genotype. SA-β-gal activity was quantified (from at least 6 mice of each genotype) using 3D Histech HistoQuant software (3DHISTECH Kft., Budapest, Hungary). Areas of SA-β-gal activity were detected based on color and their total area was divided by the total analysis area.

### SA-β-gal staining

Detection of SA-β-Gal activity was performed on frozen sections of liver tissue or cells in culture. Frozen sections or cells were fixed with 0.5% gluteraldehyde in PBS for 15 min, washed with PBS supplemented with 1 mM MgCl_2_ at pH5.5, and stained for 5–6 hours at 37°C without CO_2_ in PBS containing 1 mM MgCl_2_ at pH5.5, 1mg/ml X-Gal, and 5 mM of both potassium ferricyanide and potassium ferrocyanide. Sections were counterstained with Eosin.

### Detection and modification of gene expression

For whole livers: lysates were generated using standard RIPA buffer supplemented with phosphatase inhibitors and protease inhibitors (both from Sigma). Detection of protein expression by immunoblotting in whole liver lysates was performed using anti-αSMA (Santa Cruz), anti-β-Tubulin (Sigma). For quantitative RT–PCR total RNA was isolated using NucleoSpin kit (Macherey Nagel, Düren, Germany) and 2 μg was reverse transcribed using the RevertAid H Minus First Strand cDNA synthesis Kit (Fermentas, Glen Burnie, MD, USA). The cDNA samples were amplified using Fast SYBR Green Master Mix in StepOnePlus Real-Time PCR System (both Applied Biosystems, Foster City, CA, USA). The relative expression of αSMA was normalized using the expression levels of GAPDH.

For cell culture cells: lysates were generated using standard RIPA buffer supplemented with phosphatase inhibitors and protease inhibitors (both from Sigma). Detection of protein expression by immunoblotting in cell lysates were performed using anti-p-ERK (Sigma), anti-Total ERK (Sigma),β-Tubulin (Santa Cruz, CA, USA) and GAPDH (Millipore, MA, USA). Total RNA from cells was isolated with NucleoSpin RNA II kit (Macherey-Nagel, Germany). In all, 1 μg of RNA was reverse transcribed using MMLV reverse transcriptase (Promega, USA) and random hexamer primers (Amersham – GE Healthcare, USA). qRT-PCR was performed using SYBR Green Master Mix (Applied Biosystems, USA) in a StepOnePlus instrument (Applied Biosystems, USA). ON-TARGETplus SMARTpool small-interfering RNA targeting MICA and ULBP2, and the nontargeting (control) pool were transfected into growing IMR-90 cells with Dharmafect 1 reagent (all from Dharmacon, Lafayette, CO, USA). Subsequently, cells were induced to senesce by Etoposide treatment.

To inhibit ERK activity, senescent cells were incubated in the presence of 10μM of either the MEK inhibitors: of PD184352 (Sigma), AZD6244 (Axon Medchem, VA, USA) or Trametinib (GSK1120212) (Selleck Chemicals, Boston, USA) for 24, 48, 72 or 96 hr as indicated. To inhibit ATM activity growing cells were incubated in the presence of 10μM KU60019 (R&D Systems, USA) for 1 hr or 5mM caffeine (Sigma) and then treated with 100μM Etoposide for 24 hr. to inhibit ATM activity in senescent cells 10μM KU60019 (R&D Systems, USA) was add for 24, 48, 72 and 96 hr as indicated. All inhibitors were replenished daily. Subsequently total RNA was isolated and qRT-PCR was performed as described above. Primer list is available upon request.

### Image Stream Flow cytometry

Senescent or growing IMR-90 were harvested with TryplE (Gibco, Life Technologies, USA), collected with DMEM and centrifuged with the addition of EDTA 5mM. After a wash with cold PBS and transfer through mesh, cells were labeled with 10μg/ml of αMICA or αULBP2 in 200μl of FACS buffer (5% FCS in PBS), on ice for one hour. Next cells were washed once with cold PBS and fluorescently labeled by a secondary α-mouse antibody (Jackson Immunoresearch, USA) at a concentration of 1:200 in 200μl in FACS buffer for one hour on ice. At the end of incubation cells were washed twice with cold PBS and resuspended with DAPI 1:10,000 in FACS buffer to label dead cells. Finally, cells were imaged in an ImageStreamX system and analysis was done using the amnis IDEAS software package.

### Immunofluorescence (IF)

IMR-90, WI38 and BJ cells were grown on 0.1% gelatin-coated coverslips. Subsequently, cells were blocked with human IgG and co-incubated with 10μg/ml of antibodies against either MICA, ULBP2 and isotype control for 1 hour at 4°C. then, the coverslips were washed with PBS, incubated with anti-mouse Alexa 549-conjugated secondary antibodies in PBS for 1 h at room temperature, washed again with PBS, fixed with 1% PFA for 15 minutes, and washed again in PBS containing DAPI and mounted in DAKO mounting medium. Fluorescence was detected using a Zeiss Axioscope II fluorescent microscope and image acquisition was conducted with simple PCI software.

### Statistical analysis

Data are expressed as mean + S.E. Statistical evaluation was carried out using Student's t test (two-tailed) to test for differences between the control and experimental results. Values of p<0.05 were considered statistically significant.

## SUPPLEMENTAL MATERIAL FIGURES


