# Supplementary material for: NKG2D ligands mediate immunosurveillance of senescent cells
Source: Aging (Albany NY). 2016 Feb 13;8(2):328–44. doi: 10.18632/aging.100897 (PMC4789586; doi:10.18632/aging.100897)
Supplement: Supplementary file 1 [file aging-08-328-s001.pdf]

Sagiv et al.

Supplemental Material

Supplemental figures:

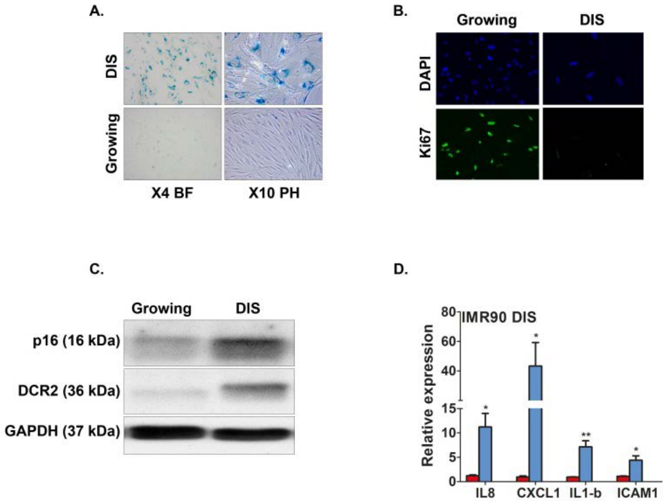

Fig S1. Identification and validation of induction of senescence by different stimuli. (A) DIS IMR-90 cells elevate SA-β-gal activity. Growing IMR-90 cells were treated with Etoposide (100μM) for 48 hours and subsequently maintained in culture for additional 7 days. Representative SA-β-gal staining confirmed elevated activity in these DIS cells compared to growing (control) cells. Images are shown in bright field and phase to observe changes in cell morphology in addition to staining. (B) DIS cells do not proliferate. DIS IMR-90 cells show lack of the proliferation marker Ki67 (Green). DAPI is shown in blue. (C) DIS IMR-90 cells elevate senescence markers. Immunoblot analysis was performed on lysates from growing and DIS IMR-90 cells. The senescence markers p16 and DCR2 are shown. GAPDH served as loading control. (D) DIS IMR90 cells elevate cytokine/chemokine expression and the adhesion molecule ICAM1 as determined by RT-PCR. Data presented as mean with S.E.M of three independent experiments. Two-tailed t-test *P<0.05, **P<0.001, ***P<0.0001.

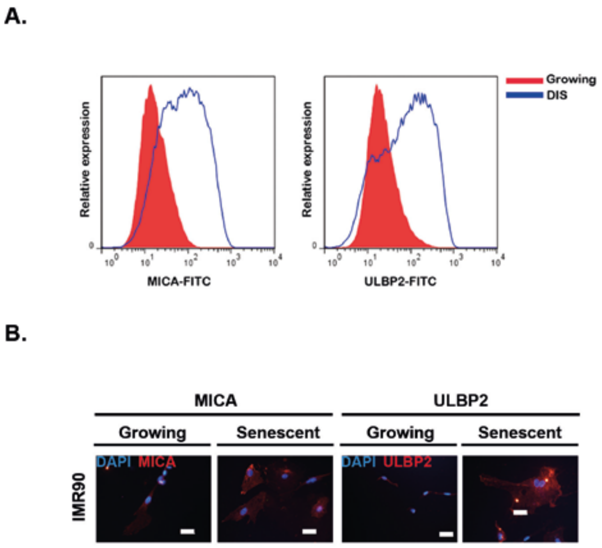

Fig S2. Immunofluorescent staining demonstrates elevated MICA and ULBP2 on the cell surface membrane of DIS IMR-90 cells. (A) Flow cytometry analysis of MICA and ULBP2 was performed on DIS IMR-90 cells to further demonstrate an elevation in MICA and ULBP2 compared to growing (control) cells. (B) Representative IF of MICA and ULBP2 confirmed higher expression levels on the cell surface membrane in DIS IMR-90 cells compared to growing (control) cells. MICA and ULBP2 are shown in red. DAPI is shown in blue.

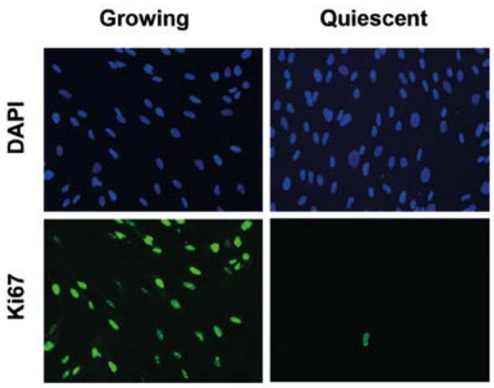

Fig S3. Validation of quiescence in IMR-90 cells by Ki67 staining. Representative image of growing cells that were grown to confluence and maintained for 7 days until cells became quiescent as verified by the lack of the proliferation marker Ki67 (Green). DAPI is shown in Blue.

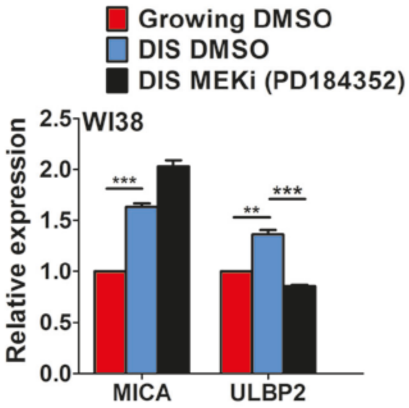

Fig S4. ERK activity contributes to ULBP2 upregulation in DIS WI38 cells. DIS WI38 cells were treated with either DMSO or 10μM of the MEK inhibitor (PD184352) for 48 hrs. Growing cells treated with DMSO served as control. Subsequently, the level of MICA and ULBP2 expression were assessed by RT-PCR, demonstrating a reduction in ULBP2 expression but not MICA. Data presented as mean with S.E.M of three independent experiments. Two-tailed t-test **P<0.001, ***P<0.0001.

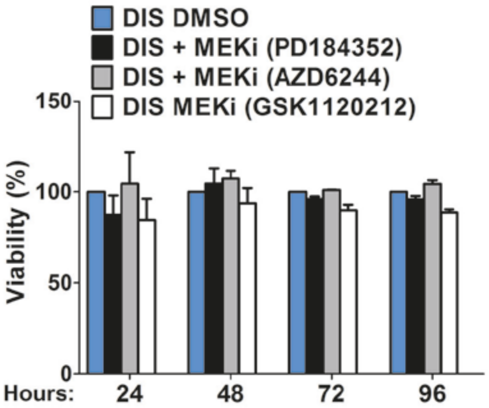

Fig S5. Inhibitors of ERK activity do not affect DIS viability. DIS IMR-90 cells were treated with either DMSO or 10μM of the MEK inhibitors: (PD184352), (AZD6244) and (GSK1120212) for 24, 48, 72 or 96 hrs and subsequently viability was assessed. No significant difference in cell viability was observed over all time points investigated. Data presented as mean with S.E.M of three independent experiments.

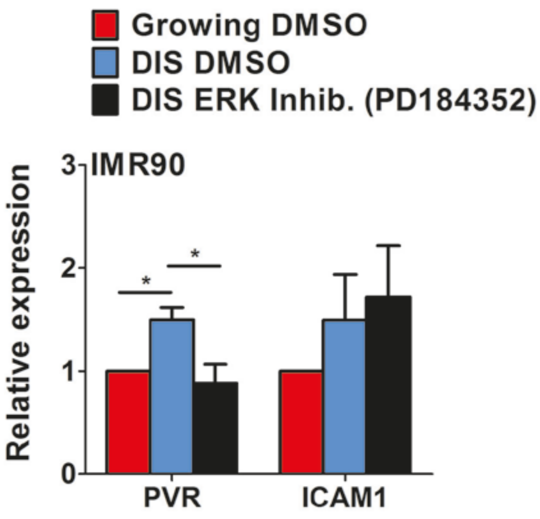

Fig S6. ERK activity contributes to PVR up-regulation but not ICAM-1 in DIS cells. DIS IMR-90 cells were treated with either DMSO or 10μM of the MEK inhibitor (PD184352) for 48 hrs. Growing cells treated with DMSO served as control. Subsequently, the level of PVR and ICAM-1 expression were assessed by RT-PCR. Data presented as mean with S.E.M of three independent experiments. Two-tailed t-test *P<0.05.

Supplementary materials & methods

1. Immunofluorescence (IF) for Ki67

For detection of Ki67 staining, growing and DNA damage-induced senescent IMR-90 were cells were plated at $5x10^4$ cells per chamber. Quiescent cells were grown to confluence and cells were maintained for a further 7 days to induce quiescence via contact inhibition. Cells were fixed in 4% for 10 minutes at room temperature, followed by permeabilization in 0.1% PBST (10 min at RT), and blocked for 1 hour in 0.1% TritonX, 15% FBS, in PBS at RT. The Ki67 antibody (Abcam, MA, USA) was diluted 1:50 in blocking buffer and incubated at 4°C overnight. Cells were washed 5 times, 10 minutes each time at RT in blocking solution on a shaker. Following Alexa488-conjugated secondary α-rabbit antibody (Jackson ImmunoResearch Laboratories, USA) was added in a 1:200 dilution in blocking buffer and incubated for 1 hour at RT in the dark. Cells were washed 5 times in 0.1% TritonX, 10 minutes each time on shaker at RT in the dark. At the end of the incubation period cells were incubated for 5 minutes in PBS containing DAPI at 1:10,000 dilution. Cells were washed twice in PBS and mounted in a fluorescent mounting media (DAKO). Immunofluorescent images were acquired on a Olympus microscope, and images were analyzed using CellP software (Diagnostic Instruments).

2. Flow cytometry

Growing or DIS IMR-90 were harvested using TryplE (Gibco, Life Technologies, USA), collected with 10% DMEM and centrifuged at 1500 RPM with the addition of

EDTA 5mM v/v. subsequently, cells were washed with cold PBS and transfer through a 100μm cell strainer. Next, $5x10^5$ were labeled with 10μg/ml of αMICA or αULBP2 (both from R&D, MN, USA) in 200μl of FACS buffer (5% FCS in PBS), on ice for one hour. Next, cells were washed once with cold PBS and re-suspended in FACS buffer containing a FITC-conjugated secondary α-mouse antibody (Jackson ImmunoResearch Laboratories, USA) and incubated for one hour on ice. At the end of the incubation period cells were washed twice with cold PBS and re-suspended with DAPI 1:10,000 in FACS buffer to label dead cells. Cells were immediately analyzed in a LSR II cytometer (BD Biosciences, Erembodegem, Belgium).

3. **Western Blot**

Lysates from growing, DIS, mcherry and mcherry-HRASv12-infected OIS IMR-90 cells were generated using standard RIPA buffer supplemented with phosphatase inhibitors and protease inhibitors (both from Sigma). Detection of protein expression by immunoblotting in cell lysates were performed using anti-p16 (Santa Cruz, CA, USA Sigma), anti-DCR , Pan-RAS and GAPDH (Millipore, MA, USA).

4. **Viability assay**

Growing and DIS IMR-90 cells were maintained in either DMSO or in the presence of 10μM of either the MEK inhibitors: of PD184352 (Sigma), AZD6244 (Axon Medchem, VA, USA) or Trametinib (GSK1120212) (Selleck Chemicals, Boston, USA) for 24, 48, 72 or 96 hr as indicated. All inhibitors were replenished daily. Subsequently cell viability was determined using Presto Blue (Life Technologies, Carlsbad, California) according to the manufacturer’s instructions.

5. RNA isolation and Real-Time PCR analysis:

Growing and DIS IMR-90 and WI38 were treated with either DMSO or 10µM of the MEK inhibitor PD184352 (Sigma) for 4 hr. subsequently, cells were harvested and total RNA from cells was isolated with NucleoSpin RNA II kit (Macherey-Nagel, Germany). 1 µg of RNA was reverse transcribed using MMLV reverse transcriptase (Promega, USA) and random hexamer primers (Amersham – GE Healthcare, USA). qRT-PCR was performed using SYBR Green Master Mix (Applied Biosystems, USA) in a StepOnePlus instrument (Applied Biosystems, USA). The following primers were analyzed:

Name	Forward	Reverse
PVR	CATCAAGCCAATACCGTGTG	TCCTGCCTGGTAGCTGATCT
ICAM-1	CAAGGCCTCAGTCAGTGTGA	GTGTCTCCTGGCTCTGGTTC
IL-1b	GCTGCTCTGGGATTCTCTTC	TGGCGAGCTCAGGTACTTC
